# Supplementary material for: Concurrent and future risk of endometrial cancer in women with endometrial hyperplasia: A systematic review and meta-analysis
Source: PLoS One. 2020 Apr 28;15(4):e0232231. doi: 10.1371/journal.pone.0232231 (PMC7188276; doi:10.1371/journal.pone.0232231)
Supplement: S1 Table — EH- endometrial hyperplasia, EC- endometrial cancer, AH- atypical hyperplasia, SH-simple hyperplasia, CAH- complex atypical hyperplasia, D&C- dilation and curettage, SAH- simple atypical hyperplasia, CH-complex hyperplasia, TVUS- transvaginal ultrasound scan, NAH- non-atypical hyperplasia. aMean age includes 8 patients diagnosed with endometrial cancer. bLikely includes n = 17 AH patients included in Agostini (2003) study. c Endometrial cancer rate per 1000 person-years was 9.3. d Follow-up time is for 249 hyperplasia patients who did not undergo hysterectomy initially. (DOCX) [file pone.0232231.s002.docx]

| **S1 Table: Characteristics of studies which assessed endometrial cancer in women with endometrial hyperplasia but unclear if cancer assessed within or after 3 months of hyperplasia diagnosis (n=21)** | | | | | | | | | | | |
| --- | --- | --- | --- | --- | --- | --- | --- | --- | --- | --- | --- |
| Author, Year  Location | Study population | Study design | Recruitment period | No. EH cases | No EC cases | % concurrent EC | Age (mean)  (SD) | Method of initial investigation | Method of follow-up investigation | Time btw biopsy and hysterectomy | EH investigated |
| Baak 2005,  Europe/ USA | 6 European centers, 2 US centers | Multicenter prospective cohort | Not reported | 197 (70 SH, 43 CH, 25 SAH, 59 CAH) | 43 (3 SH, 4CH, 5 SAH, 31 CAH) | 21.8 | Not reported | Biopsy, D&C | Not reported | Within 1 year | All |
| Byun, 2015  Korea | Busan Paik Hospital, | Single-center case-control | 2005-2013 | 117 (83 SH, 2 SAH, 14 CH, 18 CAH) | 8 | 6.8 | 46.8 (10)^a^ | Biopsy, D&C, TVUS | Hysterectomy | Not reported | All |
| Chen, 2012 Taiwan | Taiwanese Gynecologic Oncology Group | Multi-center retrospective review | 1991-2009 | 386 | 125 | 32.4 | 48.9 | D&C, pipelle biopsy, hysteroscopy | Hysterectomy | 54.9 months  (median) | All |
| Daud, 2011  UK | Ipswich Hospital NHS Trust | Single-center retrospective cohort | 1998-2009 | 280 | 41 | 14.6 | 55.7 | Pipelle biopsy, D&C, hysterectomy | Hysterectomy | 2 months (median, range 2 weeks-3years) | All |
| Dunton,1995 USA | Thomas Jefferson University Hospital | Single-center retrospective cohort | 1989-1993 | 45 (18 SH, 4 CH, 23 CAH) | 12 (CAH) | 26.7 | 51.2 (SH)  53 (CH)  57.4 (CAH) | Biopsy, D&C, hysteroscopy | Hysterectomy | 2.4 months (mean, range 1-12 months) | All |
| Giede, 2008 Canada | Saskatoon Health Region | Multicenter retrospective cohort | 2001-2006 | 70 | 25 | 35.7 | 57.4 (10.8) | Biopsy, D&C | Hysterectomy | 78 days  (mean) | AH |
| Janicek, 1993  USA | Johns Hopkins Hospital, Maryland | Single-center retrospective cohort | 1989-1992 | 44 (14 SAH, 30 CAH) | 19 | 43 | 55.5 | Biopsy, D&C | Hysterectomy | 10 weeks  (mean) | AH |
| Jesadapatrakul, 2005  Thailand | Bangkok Metropolitan Administration Medial college and Vajira Hospital | Two-center retrospective cohort | 1995-2004 | 46 (20 SH, 10 CH, 1 SAH, 15 CAH) | 8 (CAH) | 17.4 | 49.9 (9.1) | D&C | Hysterectomy | 6.7 weeks (median, range 1.4-35.9) | All |
| Kurosawa, 2012  Japan | Tohoku University Hospital, Sendai | Single-center retrospective cohort | 2001-2011 | 22 | 9 | 36.4 | 53.4 years (9.1) (EC cases)  53.4 (8.3) (EC non-cases) | Hysteroscopy, D&C | Hysterectomy | 1.9 months  (mean, EC cases)  2.7 months (mean, non-EC cases) | AH |
| Kurt, 2012 Turkey | Aegean Maternity and Women’s Health Hospital, Karsiyaka State Hosptial | Two-center retrospective cohort | Not reported | 58 (20 SAH, 38 CAH) | 17 (CAH) | 29.3 | 51.7 (9.2) | Fractional curettage | Hysterectomy | 4.9±1.7 weeks | SAH, CAH |
| Leitao, 2010 USA | Memorial Sloan-Kettering Cancer Center | Single-center retrospective cohort | 1994-2008 | 197 | 67 | 34 | 54  (median, range 32-86) | D&C, biopsy | Hysterectomy | 47 months (median) | CAH |
| Matsuo, 2015 USA | University of Southern California | Single-center case-control | 2003-2014 | 211 (24 SH/SAH, 58 CH, 129 CAH) | 43 ( 1 SH/SAH, 9 CH, 33 CAH) | 20.4 | 45.2 (9.1) | Pipelle biopsy, D&C, vacuum aspiration | Hysterectomy | 105 days (median) | All |
| Pivano, 2016 France^b^ | Hôspital la Conception and Hôspital Nord | Two-center retrospective cohort | 1996-2014 | 32 | 2 | 6.2 | 52.6 (8.5) | Hysteroscopy | Hysterectomy | 60 days (median, range 13-150) | AH |
| Reed, 2010 USA | Group Health plan, Washington State | Population-based retrospective cohort | 1985-1996 | 1443 (1,201 CH, 242 AH) | 71 (35 CH, 36 AH) | 4.9 | 18-88 (range) | Biopsy | Cancer registry | 5.3 years  (median, range 8 weeks -20.8 years) | CH, AH |
| Robbe, 2012 | Not reported | Retrospective cohort | 1999-2006 | 39 | 25 | 64 | 59 (median, range 34-90) | D&C, pipelle biopsy | Hysterectomy | 6 weeks (median, range 2-37) | CAH |
| Shutter, 2005 USA | Columbia University Medical Center | Single-center retrospective cohort | 1996-2003 | 60 | 29 | 48 | Not reported | Biopsy, D&C | Hysterectomy | 1 week-4 months | AH |
| Sirimusika, 2014  Thailand | Songklanagarind Hospital | Single-center retrospective cohort | 2000-2012 | 293 | 12 | 4.1 | 47 (mean, range 27-86) | Biopsy, D&C, hysteroscopy | Hysterectomy | 56.4 months (median, range  1.4-132.1)^d^ | All |
| Touboul, 2014 France | Tenon and Bichat Tertiary University Centers | Single-center retrospective cohort | 2002-2012 | 79 | 19 | 24 | 60.1 (11.2) | Biopsy, hysteroscopy, D&C | Hysterectomy | 64 days (median) | AH |
| Turan, 2012  Turkey | Etlik Zubeyde Hanim Women’s Health Research and Teaching Hospital | Single-center retrospective cohort | 1993-2010 | 125 | 77 | 61.6 | 54.4 years (range 32-83) | Pipelle biopsy, D&C | Hysterectomy | 20 days (median, range 5-152) | CAH |
| Yang, 2012 China | Shilong People’s Hospital and The People’s Hospital of Jieyang City | Two-center retrospective cohort | 2000-2011 | 139 (5 SH, 8 SAH, 41 CH, 85 CAH) | 42 (1 SAH, 2 CH, 39 CAH) | 30.2 | 37-69 (range) | Biopsy | Hysterectomy | Within 1 year | All |
| Zhou, 2014  China | Shanghai First Maternity and Infant Hospital | Single-center retrospective cohort | 2008-2013 | 149 | 98 | 65.8 | 49.3 years (AH to AEH group)  55.9 years (AH to EC group) | D&C, hysteroscopy | Hysterectomy | Within 6 months | AH |

EH- endometrial hyperplasia, EC- endometrial cancer, AH- atypical hyperplasia, SH-simple hyperplasia, CAH- complex atypical hyperplasia, D&C- dilation and curettage, SAH- simple atypical hyperplasia, CH-complex hyperplasia, TVUS- transvaginal ultrasound scan, NAH- non-atypical hyperplasia

^a^Mean age includes 8 patients diagnosed with endometrial cancer

^b^Likely includes n=17 AH patients included in Agostini (2003) study

^c^ Endometrial cancer rate per 1000 person-years was 9.3

^d^ Follow-up time is for 249 hyperplasia patients who did not undergo hysterectomy initially
