# Supplementary material for: Concurrent and future risk of endometrial cancer in women with endometrial hyperplasia: A systematic review and meta-analysis
Source: PLoS One. 2020 Apr 28;15(4):e0232231. doi: 10.1371/journal.pone.0232231 (PMC7188276; doi:10.1371/journal.pone.0232231)
Supplement: S2 Table — EH- endometrial hyperplasia, EC- endometrial cancer, AH- atypical hyperplasia, SH-simple hyperplasia, CAH- complex atypical hyperplasia, D&C- dilation and curettage, SAH- simple atypical hyperplasia, CH-complex hyperplasia, NAH- non-atypical hyperplasia. (DOCX) [file pone.0232231.s003.docx]

| **S2 Table: Characteristics of studies which assessed the prevalence of concurrent endometrial cancer in women with endometrial hyperplasia which did not report time between biopsy and hysterectomy** **(n=23)** | | | | | | | | | | |
| --- | --- | --- | --- | --- | --- | --- | --- | --- | --- | --- |
| Author, Year  Location | Study population | Study design | Recruitment period | No. EH cases | No EC cases | % concurrent EC | Age (mean) | Method of initial investigation | Method of follow-up investigation | EH investigated |
| Antonsen, 2011 Denmark | Danish Gynaecological Cancer Database | Population-based retrospective cohort | 2005-2010 | 773 | 457 | 59.1 | 63  (range 25-98) | Pipelle biopsy, D&C, hysteroscopy | Hysterectomy | AH |
| Brownfoot, 2014  Australia | Royal Women’s Hospital, Melbourne | Single-center retrospective cohort | 1992-2012 | 92 | 21 | 22.8 | Not reported | Biopsy, hysteroscopy | Hysterectomy | CAH |
| Costales, 2014 USA | University Texas MD Anderson Cancer Center | Single-center retrospective cohort | 1995-2013 | 150 | 55 | 36.7 (range 27-89) | 55 | Biopsy, D&C | Hysterectomy | CAH |
| Eddib, 2012 | Buffalo General Hospital and Millard Fillmore Suburban Hospital | Single-center retrospective cohort | 1999-2006 | 66 | 11 | 17 | 60±9 | Biopsy, D&C | Hysterectomy | CAH |
| Gundem 2003,  Turkey | Ege University Hospital | Single-center retrospective cohort | 1996-1999 | 103 (94 SH, 2 SAH, 5 CH, 2 CAH) | 2 | 1.9 | 48.7 years (range 36-78) | D&C | Hysterectomy | All |
| Hunter, 1994  USA | University of Kentucky Medical Center | Single-center retrospective cohort | 1970-1992 | 136 | 19 (AH) | 14 | 52 years (range 21-84) | Biopsy, D&C, | Hysterectomy | All |
| Hosseini Nasab, 2018  USA | Ben Taub General Hospital | Single-center retrospective cohort | 2000-2010 | 56 | 32 | 57.1 | 49.5 (11.4) | Biopsy | Hysterectomy | CAH |
| Joiner, 2015  USA | University of Arkansas | Single-center retrospective cohort | 2005-2013 | 67 (7 SH, 25 CH, 25 CAH) | 13 | 19.4 | Not reported | D&C, curettage | Hysterectomy | SH, CH, CAH |
| Kisielewski, 2016  Poland | Medical University of Warsaw | Single-center retrospective cohort | 2007-2014 | 44 | 15 | 34.1 | Not reported | Biopsy | Hysterectomy | AH |
| Lambert, 1994  Canada | Hôtel-Dieu Hospital | Single-center retrospective cohort | 1980-1991 | 66 (37 CH, 29 CAH) | 15 | 22.7 | 56.8 (CH), 57.5 (CAH) | Not reported | Hysterectomy | CH |
| Miller, 2008 USA | Walter Reed Medical Center, Brooke Army Medical Center | Two-center retrospective cohort | 1998-2005 | 78 | 36 | 46 | Not reported | Biopsy | Hysterectomy | AH |
| Mittal, 2009 USA | New York University Medical Center,  North Broward Medical Center, Thomas Jefferson University Hospital | Multicenter retrospective cohort | 2003-2006 | 87 | 35 | 40.3 | Not reported | Biopsy, D&C | Hysterectomy | CAH |
| Montalto, 2008  UK | Kent Cancer Center | Single center retrospective cohort | 2002-2005 | 46 | 3 | 6.5 | Not reported | Biopsy | Hysterectomy | AH |
| Naaman, 2015  Israel | Kaplan Medical Center | Single-center retrospective cohort | 2000-2011 | 32 | 2 | 6.3 | 53 years (AH in polyp & endometrium)  63 years (AH in polyp only) | Hysteroscopy, D&C | Hysterectomy | AH |
| Novac, 2005 Romania | Municipal Hospital of Craiova | Single-center retrospective cohort | 2000-2004 | 1139 (110 SH, 481 SH, 19 CAH, 41 CH, 488 mixed EH) | 7 (1 SAH, 4 CAH, 2 mixed EH) | 0.61 | Not reported | D&C | Hysterectomy | All |
| Oda, 2016  Japan | University of Tokyo Hospital | Single-center retrospective cohort | 2013-2015 | 20 | 4 | 20 | 47.6 (median) | Cytology, biopsy, D&C, hysteroscopy | Hysterectomy | AH |
| Orbo, 2008  Norway | Northern Norway | Multicenter prospective cohort | Not reported | 370 | 10 | 2.7 | 51.1 years | Biopsy, D&C | Hysterectomy | All |
| Pennant, 2008  UK | John Radcliffe Hospital | Single-center retrospective cohort | Not reported | 92 | 34 | 36.9 | Not reported | Biopsy | Hysterectomy | AH |
| Stephan, 2014  USA | University of Iowa Hospitals and Clinics | Single-center retrospective cohort | 2012 | 20 | 12 | 60 | Not reported | Not reported | Hysterectomy | CAH |
| Tierney, 2014  USA | Los Angeles County + USC Medical Center | Single-center retrospective cohort | 2003-20011 | 134 | 54 | 40.3 | Not reported | Biopsy | Hysterectomy | CAH |
| Touhami, 2018  Canada | L'Hôtel-Dieu de Québec Hospital | Single-center retrospective cohort | 2010-2016 | 120 | 64 | 53.3 | 57 (range, 40–82) | Biopsy, D&C | Hysterectomy | AH |
| Ventura, 2004 | New York University Medical Center  and Bellevue Hospital Centers | Two-center retrospective cohort | 1993-2001 | 12 | 2 | 16.7 | 53.5 years | Biopsy, D&C | Hysterectomy | CAH |
| Xie, 2002  China | Women's Hospital Zhejiang University | Single-center retrospective cohort | 1992-2000 | 150 (53 SH, 11 CH, 26 SAH, 60 CAH) | 34 (1 CH, 3 SAH, 30 CAH) | 22.7 | Not reported | D&C | Hysterectomy | All |

EH- endometrial hyperplasia, EC- endometrial cancer, AH- atypical hyperplasia, SH-simple hyperplasia, CAH- complex atypical hyperplasia, D&C- dilation and curettage, SAH- simple atypical hyperplasia, CH-complex hyperplasia, TVUS- transvaginal ultrasound scan, NAH- non-atypical hyperplasia
