# Supplementary material for: Concurrent and future risk of endometrial cancer in women with endometrial hyperplasia: A systematic review and meta-analysis
Source: PLoS One. 2020 Apr 28;15(4):e0232231. doi: 10.1371/journal.pone.0232231 (PMC7188276; doi:10.1371/journal.pone.0232231)
Supplement: S3 Table — aPremenopausal women. b Postmenopausal women. cLNG-IUS (levonorgestrel intrauterine system) treated group. d Oral progesterone-treated group. (DOCX) [file pone.0232231.s004.docx]

| S3 Table. Summary of sub-group and sensitivity analyses for concurrent and future risk endometrial cancer in women with endometrial hyperplasia | | | | |
| --- | --- | --- | --- | --- |
|  | No of studies included | Pooled estimate  (95% CI) | I-squared | P-value |
| *Concurrent endometrial cancer* | | | | |
| Studies with quality score less than 5 | 13 | 32.2 (24.1, 41.40 | 86.5 | <0.001 |
| Studies with quality score 5 or more | 2 | 31.1 (14.1, 55.40 | 95.9 | <0.001 |
| Excluding Agostini, 2003 | 14 | 33.1 (26.0, 41.1) | 88.1 | <0.001 |
| Excluding Bilgin, 2004 | 14 | 32.7 (25.4, 41.0) | 88.3 | <0.001 |
| Excluding Dolanbay, 2015 | 14 | 31.0 (23.8, 39.2) | 87.9 | <0.001 |
| Excluding Gungorduk, 2014 | 14 | 30.6 (23.8, 38.5) | 86.2 | <0.001 |
| Excluding Karamursel, 2005 | 14 | 33.3 (26.0, 41.4) | 86.1 | <0.001 |
| Excluding Kimura, 2003 | 14 | 32.4 (25.1, 40.7) | 88.4 | <0.001 |
| Excluding Lai, 2014 | 14 | 32.9 (25.5, 41.2) | 88.1 | <0.001 |
| Excluding Hahn, 2010 | 14 | 34.7 (27.9, 42.1) | 84.5 | <0.001 |
| Excluding Merisio, 2005 | 14 | 31.3 (24.0, 39.7) | 88.3 | <0.001 |
| Excluding Morotti, 2012 | 14 | 30.7 (23.7, 38.7) | 87.5 | <0.001 |
| Excluding Mutter, 2008 | 14 | 31.1 (23.5, 39.9) | 87.5 | <0.001 |
| Excluding Pavlakis, 2010 | 14 | 31.5 (24.1, 40.0) | 88.4 | <0.001 |
| Excluding Rakha, 2012 | 14 | 32.5 (24.9, 41.2) | 87.7 | <0.001 |
| Excluding Salman, 2010 | 14 | 33.2 (25.9, 41.4) | 87.9 | <0.001 |
| Excluding Valenzuela, 2003 | 14 | 31.0 (24.0, 39.1) | 88.02 | <0.001 |
| *Future risk of endometrial cancer (% per year)* | | | | |
| Studies with quality score less than 5 | 3 | 6.63 (1.23, 33.86) | 94.3 | 0.71 |
| Studies with quality score 5 or more | 7 | 2.34 (1.03, 5.31) | 86.3 | <0.001 |
| Excluding Baak, 2005 | 9 | 3.40 (1.47, 7.86) | 89.3 | <0.001 |
| Excluding Brownfoot, 2014^a^ | 9 | 2.51 (1.22, 5.16) | 87.8 | <0.001 |
| Excluding Brownfoot, 2014^b^ |  | 2.56 (1.27, 6.00) | 89.9 | <0.001 |
| Excluding Edris, 2007 | 9 | 3.28 (1.51, 7.16) | 91.0 | <0.001 |
| Excluding Gallos, 2013^c^ | 9 | 3.76 (1.78, 7.94) | 89.0 | <0.001 |
| Excluding Gallos, 2013^d^ |  | 3.61 (1.64, 7.86) | 90.2 | <0.001 |
| Excluding Garuti, 2005 | 9 | 3.14 (1.42, 6.94) | 91.0 | <0.001 |
| Excluding Gonthier, 2015 | 9 | 2.86 (1.28, 6.38) | 90.1 | <0.001 |
| Excluding Horn, 2004 | 9 | 2.92 (1.31, 6.53) | 90.8 | <0.001 |
| Excluding Mentrikoski , 2012 | 9 | 2.73 (1.26, 5.92) | 90.3 | <0.001 |
| Excluding Steinbakk , 2011 | 9 | 3.33 (1.43, 7.71) | 90.7 | <0.001 |
| Excluding Tierney , 2014 | 9 | 2.50 (1.27, 4.92) | 86.5 | <0.001 |

^a^ Premenopausal women

^b^ Postmenopausal women

^c^LNG-IUS (levonorgestrel intrauterine system) treated group

^d^ Oral progesterone-treated group
