# Supplementary material for: Concurrent and future risk of endometrial cancer in women with endometrial hyperplasia: A systematic review and meta-analysis
Source: PLoS One. 2020 Apr 28;15(4):e0232231. doi: 10.1371/journal.pone.0232231 (PMC7188276; doi:10.1371/journal.pone.0232231)
Supplement: S1 Appendix — (DOCX) [file pone.0232231.s005.docx]

**Appendix S1: MEDLINE search strategy**

| 1. Endometrial Hyperplasia/ |  |
| --- | --- |
|  |  |

| 2. "simple endometrial hyperplasia".mp. [mp=title, abstract, original title, name of substance word, subject heading word, keyword heading word, protocol supplementary concept word, rare disease supplementary concept word, unique identifier] |  |
| --- | --- |

| 3. "complex endometrial hyperplasia".mp. [mp=title, abstract, original title, name of substance word, subject heading word, keyword heading word, protocol supplementary concept word, rare disease supplementary concept word, unique identifier] |  |
| --- | --- |

| 4. "complex atypical endometrial hyperplasia".mp. [mp=title, abstract, original title, name of substance word, subject heading word, keyword heading word, protocol supplementary concept word, rare disease supplementary concept word, unique identifier] |  |
| --- | --- |

| 5. "simple atypical endometrial hyperplasia".mp. [mp=title, abstract, original title, name of substance word, subject heading word, keyword heading word, protocol supplementary concept word, rare disease supplementary concept word, unique identifier] |  |
| --- | --- |

| 6. "complex hyperplasia with atypia".mp. [mp=title, abstract, original title, name of substance word, subject heading word, keyword heading word, protocol supplementary concept word, rare disease supplementary concept word, unique identifier] |  |
| --- | --- |

| 7. "simple hyperplasia with atypia".mp. [mp=title, abstract, original title, name of substance word, subject heading word, keyword heading word, protocol supplementary concept word, rare disease supplementary concept word, unique identifier] |  |
| --- | --- |

| 8. 1 or 2 or 3 or 4 or 5 or 6 or 7 |  |
| --- | --- |
|  |  |

| 9. Uterine Neoplasms/ |  |
| --- | --- |
|  |  |

| 10. Endometrial Neoplasms/ |  |
| --- | --- |
|  |  |

| 11. Endometrial Cancer/ |  |
| --- | --- |
|  |  |

| 12. Uterine Cancer/ |  |
| --- | --- |
|  |  |

| 13. "womb cancer*".mp. [mp=title, abstract, original title, name of substance word, subject heading word, keyword heading word, protocol supplementary concept word, rare disease supplementary concept word, unique identifier] |  |
| --- | --- |

| 14. "womb neoplasm*".mp. [mp=title, abstract, original title, name of substance word, subject heading word, keyword heading word, protocol supplementary concept word, rare disease supplementary concept word, unique identifier] |  |
| --- | --- |

| 15. "womb tumour*".mp. [mp=title, abstract, original title, name of substance word, subject heading word, keyword heading word, protocol supplementary concept word, rare disease supplementary concept word, unique identifier] |  |
| --- | --- |

| 16. "womb tumor*".mp. [mp=title, abstract, original title, name of substance word, subject heading word, keyword heading word, protocol supplementary concept word, rare disease supplementary concept word, unique identifier] |  |
| --- | --- |

| 17. 9 or 10 or 11 or 12 or 13 or 14 or 15 or 16 |  |
| --- | --- |
|  |  |

| 18. 8 and 17 |
| --- |
| 19. limit 18 to females, humans and journal articles |
