# Supplementary figures and images for: Concurrent and future risk of endometrial cancer in women with endometrial hyperplasia: A systematic review and meta-analysis
Source: PLoS One. 2020 Apr 28;15(4):e0232231. doi: 10.1371/journal.pone.0232231 (PMC7188276; doi:10.1371/journal.pone.0232231)

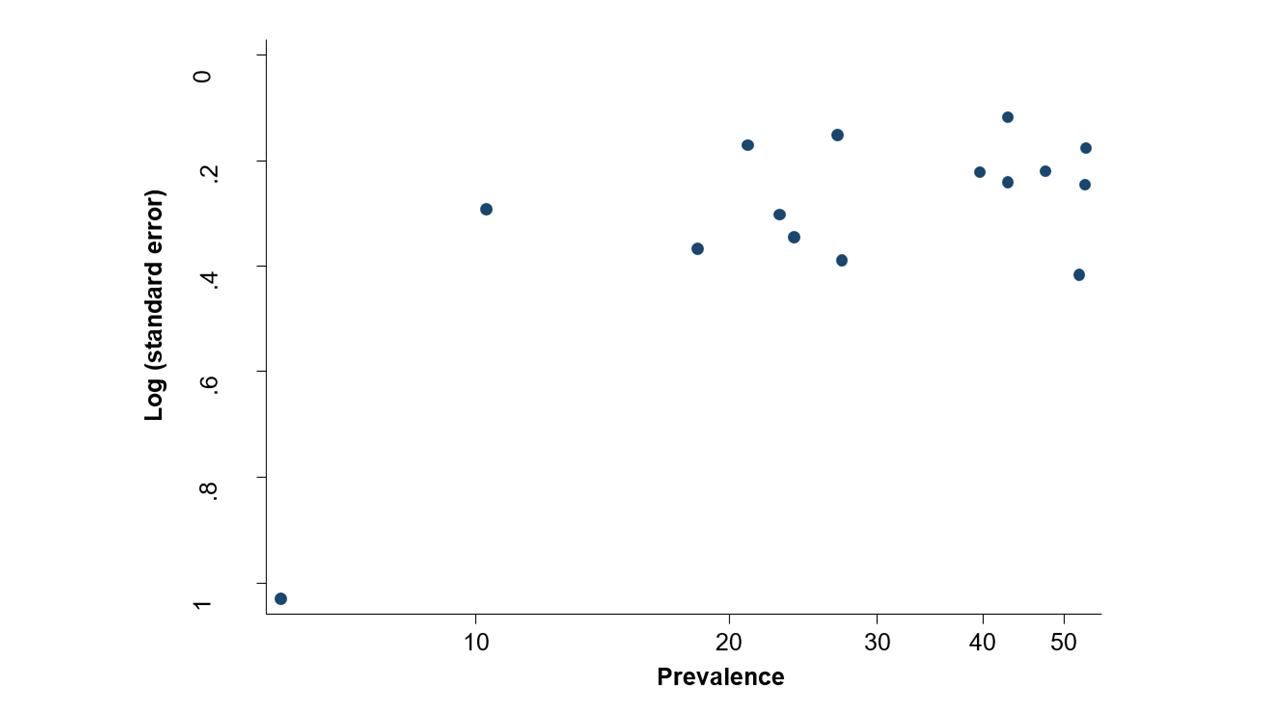

Supplement: S1 Fig — (TIF) [file pone.0232231.s006.tif]

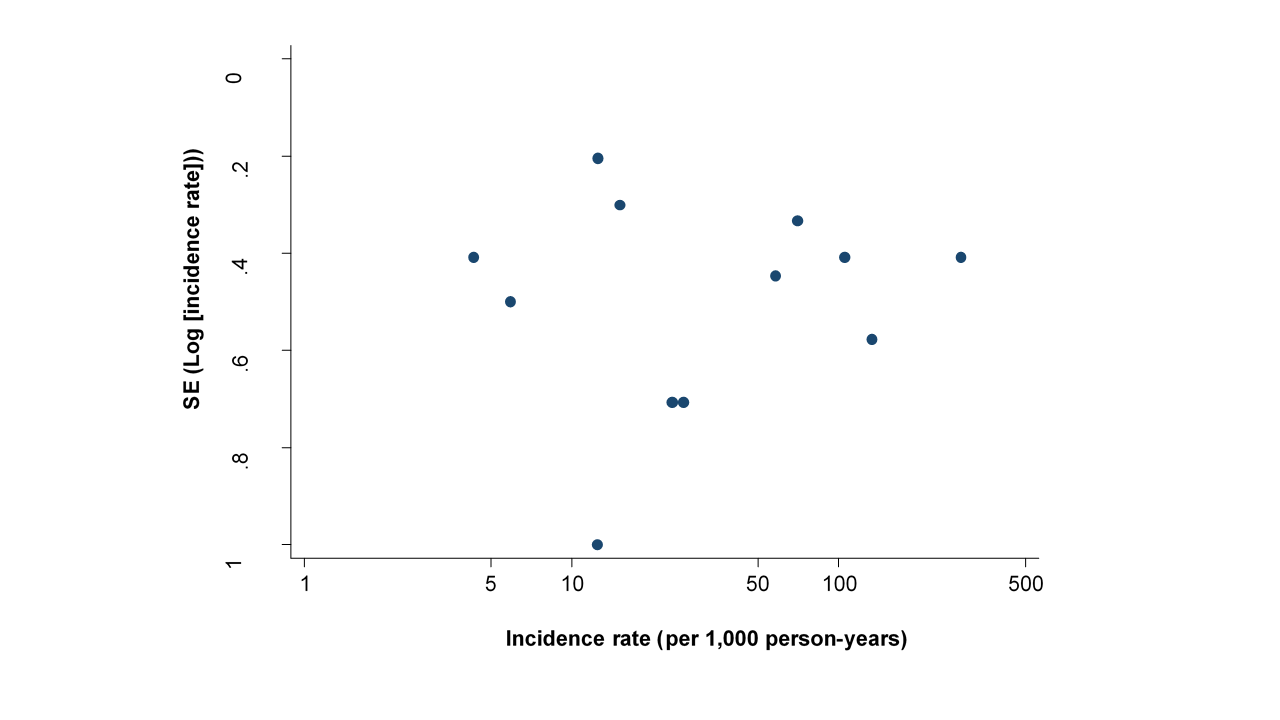

Supplement: S2 Fig — (TIF) [file pone.0232231.s007.tif]

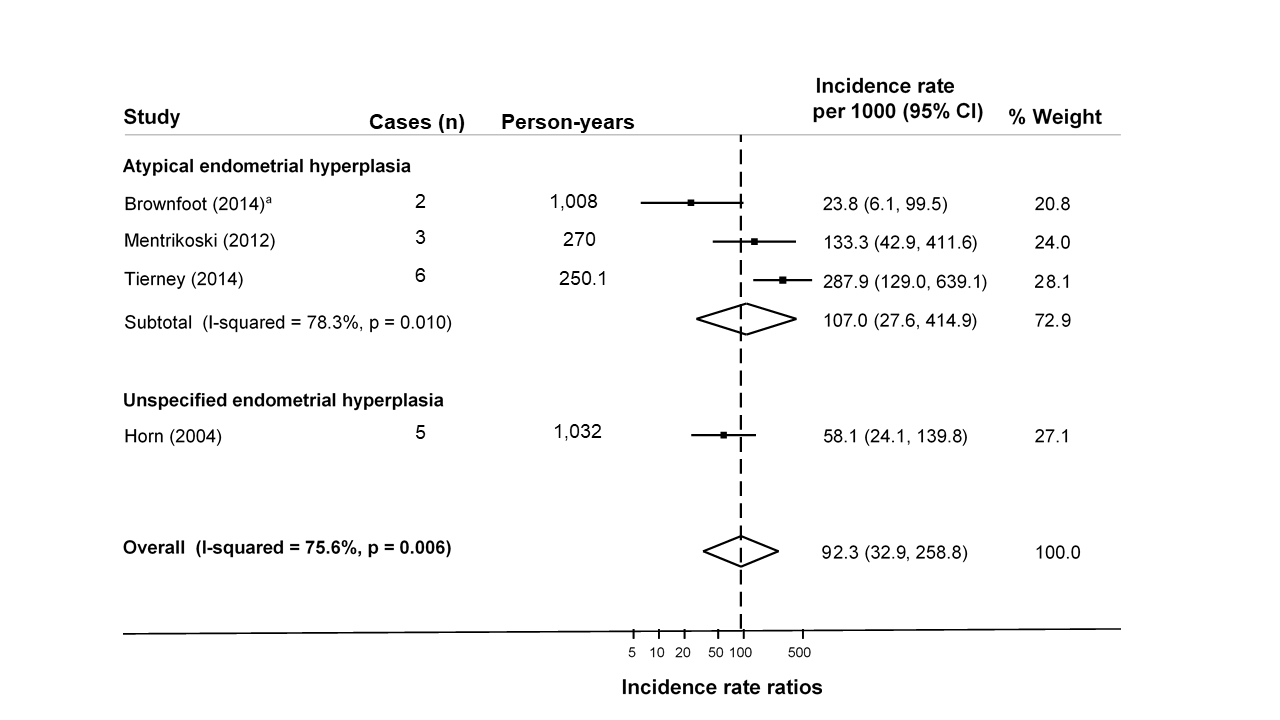

Supplement: S3 Fig — aPremenopausal women. (TIF) [file pone.0232231.s008.tif]

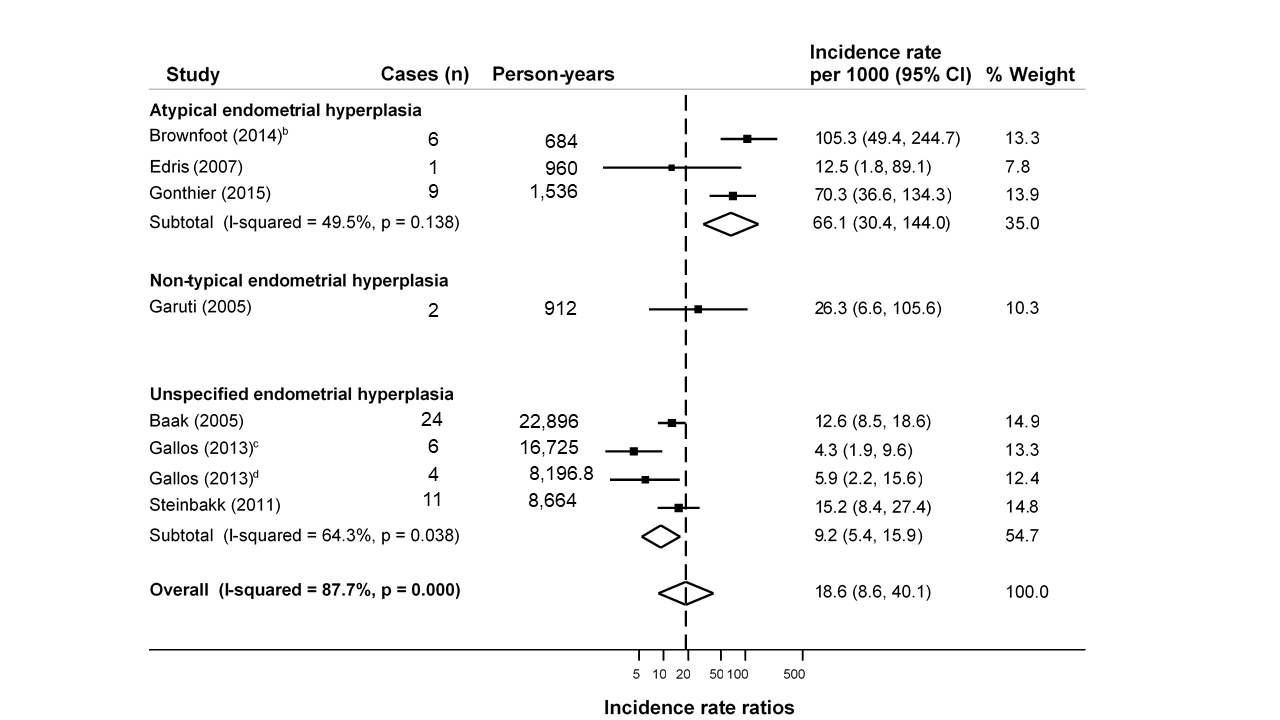

Supplement: S4 Fig — bPostmenopausal women. cLNG-IUS (levonorgestrel intrauterine system) treated group. dOral progesterone-treated group. (TIF) [file pone.0232231.s009.tif]
